# Non-Adherence to Antiseizure Medications: Rate and Predictors in Saudi Arabia

**DOI:** 10.3390/medicina60101649

**Published:** 2024-10-09

**Authors:** Noura A. Alrukban, Sarah A. Alotaibi, Layla N. Alanizy, Ahmad Saleh, Bshra A. Alsfouk

**Affiliations:** 1 Department of Pharmaceutical Sciences, College of Pharmacy, Princess Nourah bint Abdulrahman University, P.O. Box 84428, Riyadh 11671, Saudi Arabia; ph.noura.alrukban@gmail.com (N.A.A.); sarahabdualllah@gmail.com (S.A.A.); 2 Pharmacy Services Administration, King Fahad Medical City, P.O. Box 59046, Riyadh 11525, Saudi Arabia; lalanizy@kfmc.med.sa; 3 Research Center, King Fahad Medical City, P.O. Box 59046, Riyadh 11525, Saudi Arabia; asaleh@kfmc.med.sa

**Keywords:** antiepileptic drugs, compliance, epilepsy, Saudi Arabia, seizures

## Abstract

*Background and Objectives*: The objective of this paper is to determine the rate and predictors of non-adherence to antiseizure medications in Saudi Arabia. *Materials and Methods*: A cross-sectional study which involved questionnaires and data collection from patients’ medical records was conducted at neurology clinics. The rate of non-adherence to antiseizure medications was measured using “the Medication Adherence Rating Scale” (MARS). Predictors of non-adherence to antiseizure medications were evaluated using a multidimensional questionnaire specific to epilepsy. *Results*: One hundred and sixty-two patients participated in the study. The mean (SD) age was 34.1 (10.4) years, and 56% were male. Epilepsy was controlled (i.e., seizure-free ≥ 1 year) in 42% of patients. The mean ± SD (range) MARS scores were 7.80 ± 1.59 (2–10). Out of 162 patients, 58 (36%) patients had MARS scores ≤ 7 out of 10. The most frequently rated predictor for non-adherence was poor seizure control, which was reported by around 36% of patients. Forgetfulness, dosing frequency, and social stigma were also among the commonest predictors of non-adherence to antiseizure medications that were rated by approximately 27%, 24%, and 22% of the patients, respectively. The impacts of several socio-demographic and clinical factors on adherence were assessed. In the regression analysis, the odds of non-adherence in a patient who experienced adverse effects were twice that of a patient who did not have adverse effects (*p* = 0.113). Furthermore, females, employers, and patients who had comorbidity, those with focal epilepsy, those on polytherapy of antiseizure medication, and those receiving multiple doses per day, were all more likely (but not significantly, *p* > 0.05) to be non-adherent compared to their counterparts. *Conclusions*: The significance of this study is that it reveals that adherence to antiseizure medications is suboptimal in Saudi Arabia. Poor seizure control, forgetfulness, dosing frequency, and social stigma were the primary patient-reported predictors of non-adherence in epilepsy. This emphasizes the importance of routine evaluation of adherence in practice to identify and address what individual patients perceive as a barrier to adherence with antiseizure medications.

## 1. Introduction

Epilepsy is a common neurological disorder with a prevalence of around 50 million persons worldwide [1] and 3.96 per 1000 people in Saudi Arabia [2]. Epilepsy is characterized by recurring seizures that occur because of a transient imbalance in neuronal activity [3]. Antiseizure medications (ASMs) are the main treatment to control epileptic seizures. However, surgical interventions are reserved for patients with refractory epilepsy who have not responded to ASMs [4].

Non-adherence to ASMs can result in many negative consequences, including poor seizure control, status epilepticus, sudden unexpected death in epilepsy (SUDEP), hospitalization, and increased health care costs [5,6,7,8,9,10]. Additionally, poor adherence to ASMs can also make the identification of refractory epilepsy more difficult or even lead to a false identification of refractory epilepsy, which is known as pseudo-refractory [11,12].

Currently, health care practice is moving toward providing patient-centered medical care by considering individual patients and involving them in clinical decisions. To align the current approach, utilizing a standardized patient-administered questionnaire for adherence assessment can help in identifying adherence barriers and then formulating a customized solution either through educational, behavioral, or mixed strategies [13,14] which will ultimately improve treatment outcomes.

Non-adherence to ASMs is a common problem in patients with epilepsy with an estimated rate of up to 65% [15,16]. Medication adherence is a complex issue involving different factors that are related to patients, epilepsy, ASMs, and social support [6]. It is important to determine factors associated with non-adherence to allow clinicians to identify patients at highest risk early, understand their reasons, and manage them with more attention. There were many studies that assessed the factors associated with medication non-adherence in epilepsy. The literature shows that the established reasons for poor adherences are forgetfulness, adverse drug effects, and perceived epilepsy-related stigma [15,17,18,19,20,21,22,23,24,25]. On the other hand, there has been controversy in the literature regarding the influence of other factors affecting adherence such as dosing frequency, age, gender, seizure types, and spiritual belief. While several studies have demonstrated increased non-adherence rates with higher dosing frequency [22,26], one study found no association [27], and another study [28] showed better adherence with more frequent dosing. Regarding age, some studies identified younger age (under 30 years) as a correlating factor for non-adherence [6,10,17], while two studies [25,29] have suggested that older age was a predictor of non-adherence. However, several other studies found no association between age and adherence [15,27,30]. Likewise, several studies have reported no correlation with gender [27,30], while others found that men were associated with poor adherence [6,7,17]. With regard to seizure types, non-adherence was more frequently reported in generalized seizures compared to focal seizures in some studies [6,10], whereas no association was demonstrated in other studies [15]. A systematic review that examined the impact of religious and spiritual factors on adherence to pharmacological therapy revealed that while some studies demonstrated a positive correlation between religious/spiritual involvement and adherence, others indicated a negative or mixed effect on therapeutic adherence [31].

In some populations, including Muslims in Saudi Arabia, spiritual and religious beliefs affect medication adherence in epilepsy. However, a limited number of studies have been conducted to assess ASMs non-adherence in Saudi Arabia. There was a study in Saudi Arabia by Gabr and Shams [19] which evaluated adherence among adolescents. Another study, by Zafar et al. [20], measured adherence by asking the patients one question (whether they ever missed or stopped medication). Therefore, more studies are needed to recognize the determinants of non-adherence to ASMs by including different age groups and by using a multidimensional questionnaire. This study aims to determine the rate and predictors of non-adherence to ASMs in Saudi Arabia.

## 2. Methodology

### 2.1. Study Design and Patient Recruitment

A cross-sectional observational design was used in this study. Data were collected through patient-administered questionnaires and a review of medical records. Patients were recruited from 1st to 29th of July 2021 from the neurology clinics of King Fahad Medical City in Riyadh, Saudi Arabia. Inclusion and exclusion criteria are described below.

Inclusion criteria:

- Age 18 years or above, as the target study population was adults.
- Confirmed diagnosis of epilepsy, since the study focused on epilepsy patients. Patients prescribed ASMs for other conditions (e.g., migraines, trigeminal neuralgia, anxiety, bipolar disorder) were excluded.
- On antiseizure medications for at least 4 weeks, as the study assessed patient behavior in the last 4 weeks.

Exclusion criteria:

- Illiteracy, as the study scale was self-administered and required reading comprehension.
- Any form of intellectual disability, since participants needed to understand and accurately respond to the scale.
- Patient refusal to participate for any reason, as participation was voluntary and in accordance with ethical patient rights.

All other patients were included to ensure a representative sample of the epilepsy population. The study further aimed to assess the impact of various factors on medication adherence.

A total of 257 patients were assessed for eligibility; 162 met the criteria and were included in the study. Patients were recruited into the study using a convenient sampling technique. The patients were screened for eligibility by reviewing their medical records. The research team approached all eligible patients by phone call and social media application to obtain informed consent. The patients who agreed to participate were instructed to fill out the electronic questionnaires.

### 2.2. Adherence Measure

The rate of adherence to ASMs was measured using “the Medication Adherence Rating Scale” (MARS), Supplementary File S1. The author, Dr. Thompson, granted permission for the use of the MARS [32]. It is a valid patient-administered medication adherence scale. It includes 10 yes-or-no statements, with a corresponding “1” score for a “yes” response to questions 7 and 8, and a “no” response to the other questions. The total MARS score can range from 0 to 10.

Predictors of non-adherence to ASMs were investigated using another questionnaire which was specific to epilepsy. This questionnaire on non-adherence predictors was developed by our research team in both English and Arabic. It was constructed based on guidelines and best practices for developing and validating questionnaires [33,34]. It consists of ten statements that cover the most important predictors of non-adherence to ASMs. The selection of the questionnaire items was based on a comprehensive literature review; the WHO’s five dimensions of adherence to drugs [35]; and other surveys for measuring adherence, such as the Drug Attitude Inventory (DAI) [36], the Medication Adherence Questionnaire (MAQ) [37], and MARS [32]. The questionnaire then underwent an expert panel review to evaluate its content validity. Three independent experts judged the questionnaire using a specific evaluation form. A pre-test on a small sample of patients was conducted. The questionnaire was psychometrically evaluated, and all necessary statistical analyses for reliability and validity were performed. The reliability analysis of the questionnaire showed satisfactory internal consistency with a Cronbach’s alpha of 0.8, larger than the cut-off point (0.7) required for new questionnaire reliability. Additionally, the intraclass correlation coefficient (ICC) for the questionnaire with a 95% confidence interval (CI) showed good reliability, 0.8 (0.72 to 0.86), *p*-value < 0.001. The test–retest correlation coefficient demonstrated a strong correlation of ρ = 0.65 (*p*-value < 0.001). Principal component analyses (PCAs) were conducted to assess the questionnaire’s validity. Furthermore, a moderate positive correlation was found between the results of the questionnaire on non-adherence predictors and that of the pre-existing MARS scale [32], ρ = 0.28, *p*-value < 0.001. The study questionnaire on non-adherence predictors consists of 10 agree/disagree answerable statements.

In this study, MARS was selected to measure adherence and to validate the questionnaire on non-adherence predictors. DAI [36] and MAQ [37] are the two other most often used instruments for assessing medication adherence. Compared to DAI and MAQ, MARS provides several benefits. Because MARS includes both the attitude items from DAI and the problematic behavior items from MAQ, it has a higher validity. Moreover, MARS is a rapid and easy scale with only 10 yes/no questions, making it more clinically useful [32].

### 2.3. Procedures and Data Collection

Eligible patients who provided informed consent were instructed to fill out electronic versions of the study questionnaires based on their behavior over the last four weeks. Then, their total adherence score was calculated.

Simultaneously, socio-demographic and clinical characteristics data were gathered from patients’ electronic clinical notes using a pre-defined case report form. The data collected on socio-demographic and clinical characteristics included birth year, age, gender, marital status, residence, education status, employment status, comorbidities, and co-medications. Epilepsy-related information included epilepsy type, epilepsy duration, epilepsy status, and last seizure date. ASM-related information included ASM regimen, ASM name, dose, frequency, side effects, and the latest adherence evaluation by health care providers.

### 2.4. Study Outcomes and Variables

The primary endpoint of this study was the rate of non-adherence to ASMs, which was measured by MARS. The secondary endpoint of the present study was to identify the potential predictors of non-adherence to ASMs, which were evaluated by the second study questionnaire that was specific for epilepsy. The questionnaire domains are socio-economic, health care system-related, patient-related, and ASM-related aspects.

Comorbidity was defined in this study as a current health condition. Current comorbidities were collected and classified into nine categories based on the body’s organ systems, including vitamin and mineral deficiency, endocrine disease, cardiovascular disease, neurological and/or musculoskeletal disease, psychiatric disease, gastrointestinal disease, renal and/or hepatic disease, respiratory disease, and other comorbidities. The history of health conditions which no longer exist was not included.

Epilepsy type was categorized into generalized, focal, and unclassified according to the latest guidelines for seizure and epilepsy classification by the International League Against Epilepsy (ILAE) [38].

In this study, epilepsy status was categorized as controlled if the patient had been seizure-free for at least 12 months at data collection time and uncontrolled if not. The adverse effects of ASMs were documented based on the patients’ reports and physicians’ clinical assessments. The health care providers’ evaluation of ASMs adherence was recorded as good adherence or poor adherence depending on assessments by clinicians during routine clinical visits, as documented in the patients’ medical records.

### 2.5. Statistical Analysis

Data analyses were conducted by the Statistical Package for Social Science (SPSS version 25).

Categorical data were illustrated as frequencies and percentages, whereas continuous data are presented as mean ± standard deviation (SD) and range.

A single population proportion formula was used to calculate the target sample size. The non-adherence to ASMs population proportion was estimated at 50% from previous studies [20,39], with ±6 margin of error and an available population of patients of 400 at the 95% confidence level, providing a minimum sample size of 161.

The rate of adherence was measured by the MARS scale, which includes 10 yes-or-no statements. The total MARS score (out of 10) was calculated for each patient. The MARS scores of patients are presented as mean ± SD (range) and illustrated as a boxplot, which shows the median, interquartile range (IQR), and range of patients scores.

Predictors of non-adherence were evaluated by the second study questionnaire that was specific to epilepsy. Predictors were presented as percentages of responses (agree or disagree) to each scale item.

A multivariate logistic regression test was conducted to assess the association between potential risk factors (independent variables) and non-adherence to ASMs (dependent variable). The dependent variable was binary outcome (i.e., adherent vs. non-adherent) in the regression analysis; patients with a total scale score of ≤7 out of 10 were categorized as non-adherent while those with a score of ≥8 were classified as adherent. The adjusted odds ratio (OR) with a 95% confidence interval (95% CI) was used in regression analysis for the investigated variables. The age factor was included in the analysis as a numeric variable, while the remaining factors were binary categorical variables. The variables were entered into the regression model using the “Enter” method. A *p*-value of <0.05 was considered statistically significant.

### 2.6. Ethical Considerations and Approvals

Institutional review board (IRB) approval was obtained prior to the data collection process (IRB log Number 21-071) from the Research Ethics Committee at King Fahad Medical City, Riyadh, Saudi Arabia. The declaration of Helsinki and guidelines for good clinical practice were followed.

All included patients provided informed consent before conducting the study. It was clearly stated that all information was confidential and used for research purposes only, and their participation was completely voluntary.

The patients’ confidentiality was maintained during the study. The dataset contained no potential identifiers or personal information, and data access was limited to the research team by applying physical and IT security.

## 3. Results

### 3.1. The Demographic and Clinical Characteristics of the Patients

One hundred and sixty-two patients participated in the study. The mean (SD) age was 34.1 (10.4) years, and the range was 18–84 years. Table 1 demonstrates the patients’ characteristics: approximately 56% were male, and around 51% of the patients were married. In terms of residence, half of the patients were from outside Riyadh. Approximately 64% of the patients had higher education degrees. Unemployment was documented in about 36% of the patients, the remaining were either employed or their employment status was unknown.

Out of 162 participants, 91 (56%) had at least one chronic comorbidity, whereas 71 patients (44%) had only epilepsy with no comorbidity. As shown in Table 2, the most common comorbidities were vitamin and mineral deficiency, followed by endocrine diseases. About 53% of the included patients were on co-medications other than ASMs.

Table 3 illustrates epilepsy and ASM information. Focal epilepsy accounted for 50%. Around 25% of the patients had been diagnosed with epilepsy for longer than 21 years. Epilepsy status was considered uncontrolled in approximately 54% of patients. Furthermore, ASM regimens were used as monotherapy in about 38% of patients, the remaining patients received a combination of ASMs. Regarding ASM monotherapy, levetiracetam was the most frequently prescribed agent (*n* = 31), followed by lamotrigine (*n* = 13) and carbamazepine (*n* = 13), then topiramate (*n* = 3) and valproate (*n* = 2). Additionally, monotherapy ASM was prescribed with multiple dosing frequencies per day in the majority of patients (90%). Side effects from ASMs within the last four weeks were identified in 21% of the patients. Health care provider evaluations of ASMs adherence were not documented in 58% of the patients.

### 3.2. Rate of Adherence to ASMs

**Figure 1:**
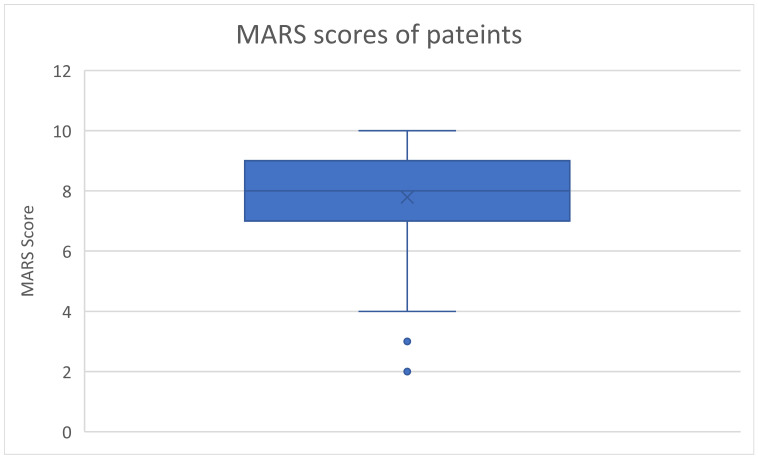
Scores on the Medication Adherence Rating Scale (MARS) of the patients (*n* = 162). Median (interquartile range, IQR) = 7.8 (7–9) and min–max = 4–10. The illustrated boxplot shows the median (the middle line of the box), IQR (the upper and lower lines of the box), min–max (the upper and lower whiskers), and outliers (the dots) of the data.

The scores of MARS of the patients are described in Figure 1. The mean ± SD (range) scores of the MARS were 7.80 ± 1.59 (2–10). Out of 162 patients, 58 (36%) patients had MARS scores ≤ 7 out of 10. Patients’ responses to each item of the Medication Adherence Rating Scale (MARS) are shown in Supplementary Table S1.

### 3.3. Predictors of Non-Adherence to ASMs

**Figure 2:**
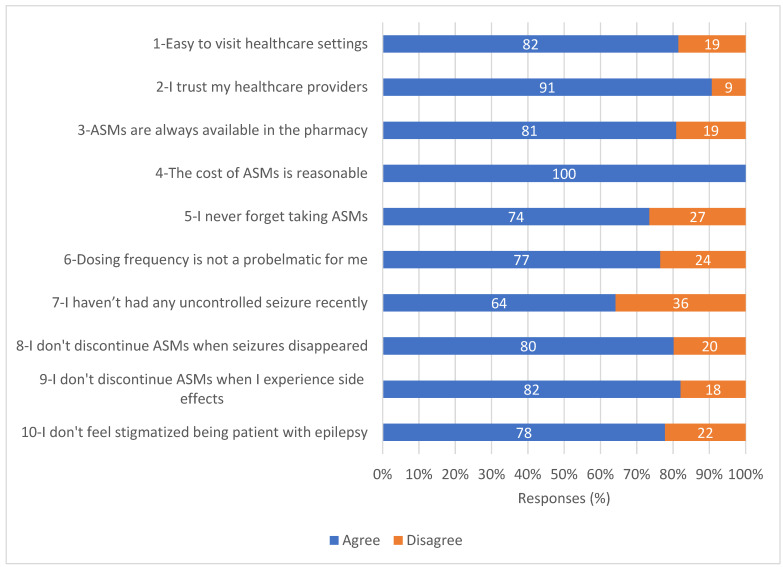
Patients’ responses (%) to the study questionnaire on potential predictors of non-adherence (*n* = 162). Abbreviation: ASMs, aAntis-Seizure mMedications.

Figure 2 shows the percentages of patients’ responses to each question of the study questionnaire. The most frequently rated predictor of non-adherence was poor seizure control by ASMs, around 36% of the patients had at least one seizure in the last four weeks. Forgetfulness, dosing frequency, and social stigma were also among the commonest predictors for non-adherence to ASMs as rated by approximately 27%, 24%, and 22% of the patients, respectively. In contrast, the majority of the patients trusted their health care providers, and only about 9% of the patients rated trust issues with health care providers as a predictor of non-adherence.

The impacts of several demographic and clinical factors on adherence to ASMs were assessed. The studied factors were the patient’s age, gender, marital status, residence, employment, presence of comorbidities, presence of psychiatric comorbidities, epilepsy type, epilepsy duration, epilepsy control by ASMs, adverse effects from ASMs, presence of ASM combinations, and whether they are administered multiple doses per day. As illustrated in Table 4, experiencing the adverse effects of ASMs was associated with an increased risk of non-adherence, but was not statistically significant. In the adjusted model of the regression analysis, the odds of non-adherence to medication in a patient suffering adverse effects from ASMs were twice that of a patient who was not having any adverse effects. Furthermore, females, employers, patients with a comorbidity or focal epilepsy, on ASM polytherapy regimens, and those receiving multiple doses per day of ASMs were more likely (but not statistically significantly so) to be non-adherent compared to their counterparts. The other investigated factors were not found to be associated with non-adherence to ASMs.

## 4. Discussion

This study aims to determine the rate and predictors of non-adherence to ASMs in Saudi Arabia. Such outcomes help clinicians identify high-risk patients early and optimize their adherence. The present study found that approximately 36% of the studied patients with epilepsy were less adherent (MARS scores ≤ 7) to the prescribed ASMs. This was a lower rate than that observed in two previous studies from Saudi Arabia; one reported a 38% non-adherence rate in adolescents [19], and another reported a 49% non-adherence rate [20] in patients of all ages. Rates of non-adherence to medication for epilepsy in other studies from different countries ranged from 28 to 66% [17,18,21,23,25,27,39,40]. This variation between studies is likely due to variation in the method of measuring adherence (objective vs. subjective measures), study settings (hospital-based vs. community-based), study design (prospective vs. retrospective), as well as variations in patients’ characteristics, health care systems, and culture and beliefs. A study in Saudi Arabia found that around 16% of patients believed that epilepsy was an untreatable condition and about 50% believed in non-medical treatments including faith and spiritual therapy [41]. Another study in Saudi Arabia showed that patients who reported using religious treatment had lower rates of adherence to medication, possibly because they placed more faith in godly healing than in the need for adequate medication adherence [42]. Although religious beliefs were not directly assessed in this study, they can affect attitudes to taking medication. Consequently, health care professionals need to become competent and sensitive about how spirituality and religion affect adherence in order to address this issue with their patients [31].

MARS evaluates medication-taking behavior, patients’ attitudes to taking medication, and adverse drug effects and attitudes to medication. Therefore, the observed non-adherence rate in this study can represent patients with actual non-adherence and those at risk for non-adherence due to adverse drug effects or negative attitudes toward taking medication.

In the current study, the most frequently rated predictor of non-adherence was poor seizure control by ASMs. This is in line with the findings of a national cross-sectional study in which a significant association between ASM non-adherence and poor seizure control (*p*-value = 0.002) was observed [20]. Poor seizure control and seizure frequency were consistently found to be associated with non-adherence to ASMs in the literature [17,19,22,40]. In fact, poor seizure control can result from inappropriate medication selection, sub-therapeutic prescribed doses, drug–drug interactions, and most importantly non-adherence to ASMs. Doubtless, even the correct medications at the correct doses cannot be effective if the patient does not take them.

If non-adherence is not recognized as a reason for the apparent medication ineffectiveness, it may lead to the unnecessary addition of another ASM or increasing the ASM dose in an attempt to manage uncontrolled seizures [22].

Forgetfulness, dosing frequency, and social stigma were also among the most commonly rated predictors for non-adherence to ASMs in the present study. Forgetting to take ASMs was the primary reason for non-adherence in previous studies [15,17,18,19,20,25]. Regarding dosing frequency, there has been controversy about the association between dosing frequency and non-adherence to ASMs [6]. Several studies have demonstrated that ASM non-adherence rates increased as the number of daily doses increased [22,26]. On the other hand, a retrospective study of 108 patients with epilepsy observed better adherence with more frequent dosing [28], which contradicts commonly held beliefs on medication adherence. It is worth noting that this study [28] had limitations as it included a small and selective cohort, and the study design was retrospective. Additionally, a cross-sectional study found no association between dosing frequency and adherence [27]. However, in this study [27], only about 6% of the included sample received a once-daily dose of ASM, which therefore affects the validity of the finding regarding the relationship between dosing frequency and non-adherence to ASMs. Regarding perceived stigma, it has been documented in the literature that feeling stigmatized is associated with lower medication adherence in epilepsy [24,43].

In the current study, the majority of the patients trusted their health care providers, and only about 9% of the patients rated trusting issues with health care providers as a predictor for non-adherence. The patient–physician relationship has been observed to be associated with medication adherence in epilepsy. A study found that adherent patients were significantly more likely to trust their doctors than non-adherent patients (34% vs. 17%, respectively) [22].

Medication adherence is a complex issue requiring health care providers to deeply understand the cause of non-adherence and what behind it. For instance, forgetfulness may result from impaired memory that may be related to epilepsy or to adverse drug effects. Patients may also forget to take their ASM because they take too many medications at different times, or due to their busy schedules. Furthermore, if non-adherence is due to difficulties in accessing health care settings for follow-up appointments or medication refills, the patient’s mobility status, and a socio-economical aspect such as the distance and cost of transportation, needed to be investigated. Patients may stop taking ASMs or reduce their dose when seizures disappear because they fail to understand the purpose and action of their medications. This emphasizes the importance of routine evaluation of patients’ adherence to medications in health care services. In fact, 58% of the patients included in this study had no recent or regular documentation of their adherence status from their health care providers.

Upon identifying a patient’s reason for non-adherence, health care providers can select the suitable intervention that address the patient’s barrier to adherence. When patients forget to take medication due to cognitive problems, using reminders such as alarms, calendars, or pill boxes is recommended [18]. In case forgetfulness due to multiple medications, health care providers can simplify the patients’ medication regimens [6]. Support from family and friends may be helpful for patients who have difficulties in accessing hospitals and pharmacies due to impaired mobility. Referral to social services is suitable if patients have socio-economic problems [6]. Patient education is a suitable intervention when failure to understand the purpose of the medication is the reason for poor medication adherence [44]. In general, interventions to improve medication adherence in epilepsy include education, consultation, a simplified dosage regimen, the use of reminders, taking ASM doses according to stable habits, and family and social support [6,14,18]. A recent systematic review of 20 randomized or quasi-randomized controlled trials found that behavioral interventions such as reminders and the use of combined interventions showed more positive effects on adherence to ASMs compared to counseling and education [14]. Consultation may include warning patients about the relationship between SUDEP and non-adherence to ASMs. A study investigating 66 SUDEP cases showed subtherapeutic or undetectable ASM plasma levels in 68% of the cases [45].

The patients who had experienced adverse effects in this study tended to be more non-adherent with their ASMs compared with those who had not suffered adverse effects. Although the difference was not statistically significant, it may have clinical significance. In fact, around 18% of the included patients admitted that they stopped taking or reduced the dose of their ASMs when their side effects interfered with their daily activities. Adverse effects have been reported to be a strong predictor of non-adherence in the literature [21,22,23,24]. This is expected because the adverse effects of ASMs affect both the physical and psychological states of patients, interfere with their daily activities, and hence affect their quality of life [46].

One of the strengths of the present study is the use of a multidimensional questionnaire specific to epilepsy with the aim of investigating predictors of non-adherence to ASMs. The construct validity of the study questionnaire was evaluated by correlating scores with the pre-existing MARS but without validation using objective methods such as drug serum levels or pill caps.

There is a potential drawback to using the patient-reported method including recall bias that may result in under- or over-reporting adherence in some cases [6]. Additionally, a medical record review was used to collect data on some variables, which may have limitations including incomplete documentation. For instance, adverse effects were collected from medical records only and not by asking patients, which may result in an underestimation of the evaluated outcomes. Studies have shown that adverse drug effects can be identified more accurately through patient reports than through medical record reviews [47]. However, there were no missing data in the primary outcomes collected by the study scales in the present study. In this study, a convenient (non-random) sampling technique was used. This sampling method is associated with selection bias. This may explain the low rate of psychiatric diseases in the study sample (*n* = 13/162). This may limit the generalizability of the study. However, convenient sampling is commonly used in clinical research because it is an easy and feasible method [33]. Furthermore, the patients were recruited from a government hospital, and therefore the cost of ASMs could not be studied as an obstacle to adherence. For future studies, we recommend expanding the questionnaire’s applicability by utilizing the tool in different settings, including private hospitals, as well as studying ASM adherence in other age groups, particularly teenagers.

In this analysis, the association between several socio-demographic and clinical factors with non-adherence was assessed. No statistically significant association was found, which could be due to the small sample size. Therefore, further large studies are necessary to investigate the exact association between socio-demographic and clinical factors with non-adherence in our region. However, the findings are potentially clinically meaningful, and we encourage consideration of such variables while evaluating ASM adherence.

In addition to evaluating medication adherence, health care providers are encouraged to assess the efficacy of medication in uncontrolled patients such as the appropriateness of ASMs and their dosages, the monitoring of adverse drug effects, and consideration of socio-economic factors such as the patient’s ability to afford treatment and access hospitals or pharmacies. This comprehensive approach offers a more complete understanding of the patient’s overall assessment.

In conclusion, adherence to ASMs plays a vital role in the management of epilepsy because non-adherence is associated with increased morbidity and mortality. This study reveals that adherence to ASMs is suboptimal in Saudi Arabia, and it also identifies common predictors of non-adherence to ASMs. Such outcomes will raise the awareness of health care providers about the importance of conducting adherence assessments regularly to identify non-adherent patients and their reasons for non-adherence, aiming to tailor an appropriate personalized intervention for an individual patient. Physicians’ awareness of medication adherence can be effectively increased through campaigns, conferences, and clinical guidelines. Overall, non-adherent patients need to be identified, understood, and managed more carefully.

## Figures and Tables

**Table 1:** Demographics information of the study patients (*n* = 162).

Characteristic	*n* (%)
Gender	
Male	91 (56.2)
Female	71 (43.8)
Marital Status	
Married	83 (51.2)
Unmarried (single or divorced)	67 (41.4)
Not documented	12 (7.4)
Residence	
Riyadh	73 (45.1)
Outside Riyadh	81 (50.0)
Not documented	8 (4.9)
Education Status	
General education	8 (4.9)
Higher education	103 (63.6)
Not documented	51 (31.5)
Employment Status	
Employed	51 (31.5)
Unemployed	59 (36.4)
Not documented	52 (32.1)

Presented data are the number of patients (*n*) and a percentage of the total number of patients (*n* = 162).

**Table 2:** Comorbidities in the study patients (*n* = 91).

Comorbidity	*N*
Vitamin and mineral deficiency	32
Endocrine disease	29
Cardiovascular disease	22
Neurological and/or musculoskeletal disease	15
Psychiatric disease	13
Gastrointestinal disease	6
Renal and/or hepatic disease	6
Respiratory disease	5
Other comorbidities	26

Presented data are the number of patients (*n*). In total, 91 patients had at least one comorbidity. The remaining 71 patients had only epilepsy with no comorbidity.

**Table 3:** Epilepsy and antiseizure medication information (*n* = 162).

Characteristic	*n* (%)
Epilepsy type	
Focal	81 (50.0)
Generalized	56 (34.6)
Unclassified	25 (15.4)
Epilepsy duration (years)	
<6	22 (13.6)
6–10	31 (19.1)
11–15	28 (17.3)
16–20	29 (17.9)
>21	40 (24.7)
Not documented	12 (7.4)
Seizure control status	
Controlled	68 (42.0)
Uncontrolled	88 (54.3)
Not documented	6 (3.7)
Antiseizure medication regimen	
Monotherapy	62 (38.3)
Dual therapy	65 (40.1)
Triple therapy or more	35 (21.6)
Dosing frequency of monotherapy	
Once daily	6 (9.7)
Multiple dosing per day	56 (90.3)
Side effect from antiseizure medication	
Yes	34 (21.0)
No	128 (79.0)
Health care provider evaluation of medication adherence	
Poor adherence	15 (9.3)
Good adherence	53 (32.7)
Not documented	94 (58.0)

Presented data are the number of patients (*n*) and a percentage to the total number of patients (*n* = 162).

**Table 4:** Multivariate logistic regression analysis for predictors of non-adherence to antiseizure medications.

Independent Variable	Adjusted OR	95% CI for OR	*p*-Value
Age (years)	0.994	0.943–1.047	0.811
Female gender	1.217	0.437–3.386	0.707
Married	0.72	0.233–2.224	0.568
Live in Riyadh	0.962	0.545–1.698	0.893
Employed	1.182	0.418–3.340	0.752
Presence of comorbidity	1.334	0.498–3.578	0.567
Presence of psychiatric comorbidity	0.867	0.137–5.482	0.879
Focal epilepsy	1.332	0.685–2.592	0.398
Epilepsy duration for longer than 15 years	0.926	0.551–1.557	0.771
Seizure-free for at least 12 months	0.999	0.374–2.665	0.998
Adverse effects of antiseizure medication(s)	2.222	0.827–5.970	0.113
Antiseizure medications polytherapy	1.107	0.418–2.932	0.839
Multiple doses per day of antiseizure medication monotherapy	1.829	0.197–16.961	0.595

CI: confidence interval, OR: odds ratio.

## Data Availability

Noura A. Alrukban and Sarah A. Alotaibi have full access to the study data. The original contributions presented in the study are included in the article, further inquiries can be directed to the corresponding authors.

## Supplementary Materials

The following supporting information can be downloaded at: https://www.mdpi.com/article/10.3390/medicina60101649/s1, Table S1: Patients’ responses on each item of Medication Adherence Rating Scale (MARS); File S1: The Medication Adherence Rating Scale (MARS).
