# Supplementary material for: Non-Adherence to Antiseizure Medications: Rate and Predictors in Saudi Arabia
Source: Medicina (Kaunas). 2024 Oct 9;60(10):1649. doi: 10.3390/medicina60101649 (PMC11509931; doi:10.3390/medicina60101649)
Supplement: Supplementary file 1 [file medicina-60-01649-s001.zip › medicina-3182515-File S1.pdf]

The Medication Adherence Rating Scale (MARS) (Thompson, Kulkarni, and Sergejew 2000)

Please respond to the following statements by circling the answer which best describes your behaviour or the attitude you have held toward your medication in the past week.

1. Do you ever forget to take your medication? Yes/No
2. Are you careless at times about taking your medicine? Yes/No
3. When you feel better, do you sometimes stop taking your medicine? Yes/No
4. Sometimes if you feel worse when you take the medicine, do you stop taking it? Yes/No
5. I take my medication only when I am sick. Yes/No
6. It is unnatural for my mind and body to be controlled by medication. Yes/No
7. My thoughts are clearer on medication. Yes/No
8. By staying on medication, I can prevent getting sick. Yes/No
9. I feel weird, like a ‘zombie’, on medication. Yes/No
10. Medication makes me feel tired and sluggish. Yes/No

Please contact the authors for additional information regarding the scale and scoring procedures at the address below:

Dr Katherine Thompson Mental Health Services for Kids and Youth, Locked Bag 10, Parkville, Victoria 3052, Australia.

Reference:

Thompson, K., J. Kulkarni, and A. A. Sergejew. 2000. 'Reliability and validity of a new Medication Adherence Rating Scale (MARS) for the psychoses', *Schizophr Res*, 42: 241-7.
