# Supplementary material for: Non-Adherence to Antiseizure Medications: Rate and Predictors in Saudi Arabia
Source: Medicina (Kaunas). 2024 Oct 9;60(10):1649. doi: 10.3390/medicina60101649 (PMC11509931; doi:10.3390/medicina60101649)
Supplement: Supplementary file 1 [file medicina-60-01649-s001.zip › medicina-3182515-Table S1.pdf]

Supplementary Table S1. Patients' responses on each item of Medication Adherence Rating Scale (MARS)

NO	MARS Question/Statement	No (n)	No (%)	Yes (n)	Yes (%)
1	Do you ever forget to take your medication?	73	45%	89	55%
2	Are you careless at times about taking your medication?	149	92%	13	8%
3	When you feel better, do you sometimes stop taking your medication?	153	94%	9	6%
4	Sometimes, if you feel worse when you take the medication, do you stop taking it?	151	93%	11	7%
5	I take my medication only when I am sick.	154	95%	8	5%
6	It is unnatural for my mind and body to be controlled by medication.	115	71%	47	29%
7	My thoughts are clearer on medication.	70	43%	92	57%
8	By staying on medication, I can prevent getting sick.	35	22%	127	78%
9	I feel weird, like a 'zombie' on medication.	144	89%	18	11%
10	Medication makes me feel tired and sluggish.	105	65%	57	35%

Adherent='No' response for q1-6,9-10 'Yes' response for q7,8
